# The Hand, Its Mechanism, and Vital Endowments, as Evincing Design

**Published:** 1834-01-01

**Authors:** 


*The Hand, its Mechanism and Vital Endowments, as evincing Design.* By Sir Charles Bell, k.g.h., &c.—London, 1833. 8vo. pp. 314.

If some whimsical nobleman, dissatisfied with "Hamlet" or "Paradise Lost," were to propose their themes to be once more sung by the poets of the day, as we are certain that nothing would be produced to rival the ancient masterpieces, so we are sure that Southey or Wordsworth would honour their patron's choice by many splendid verses; and thus, though the strange bequest of the Earl of Bridgewater, were it decupled, could not call forth another "Natural Theology," still it has been the exciting cause of several excellent treatises—and this is one of them.

We shall not attempt an analysis of the work, as it is extremely discursive, and consists of a number of essays on different points in comparative anatomy; but we shall content ourselves with presenting to our readers several extracts from various parts of the book. In the introductory chapter our author has the following beautiful observations:

"The passiveness which is natural in infancy, and the want of reflection as to the sources of enjoyment which is excusable in youth, become insensibility and ingratitude in riper years In the early stages of life, before our minds have the full power of comprehension, the objects around us serve but to excite and exercise the outward senses. But, in the maturity of reason, philosophy should present these things to us anew, with this difference, that the mind may contemplate them; that mind which is now strengthened by experience to comprehend them, and to entertain a grateful sense of them.

"It is this sense of gratitude which distinguishes man. In brutes, the attachment to offspring for a limited period is as strong as in him, but it ceases with the necessity for it. In man, on the contrary, the affections continue, become the sources of all the endearing relations of life, and the very bonds by which society is connected.

"If the child upon the parent's knee is unconsciously incurring a debt, and strong affections grow up so naturally that nothing is more universally condemned than filial ingratitude, we have but to change the object of affection, to find the natural source of religion itself. We must shew that the care of the most tender parent is in nothing to be compared with those provisions for our enjoyment and safety, which it is not only beyond the ingenuity of man to provide, but which he can hardly comprehend while he profits by them.

"If man, of all living creatures, be alone capable of gratitude, and through this sense be capable also of religion, the transition is natural; since the gratitude due to parents is abundantly more owing to Him 'who saw him in his blood, and said Live.'" (P. 8.)

In the second chapter, in which Sir Charles treats of the adaptation of the skeleton to the mode of life for which the animal is designed, he notices a curious error of Buffon and others, who threw away their mistaken compassion on some animals whose tardy motion they supposed to arise from a defective organization.

"Modern travellers express their pity for these animals. Whilst other quadrupeds, they say, range in boundless wilds, the sloth hangs suspended by his strong arms, a poor ill-formed creature, deficient as well as deformed, his hind legs too short, and his hair like withered grass; his looks, motions, and cries conspire to excite pity; and, as if this were not enough, they say that his moaning makes the tiger relent and turn away. This is not a true picture: the sloth cannot walk like quadrupeds, but he stretches out his long arms, and if he can hook on his claws to the inequalities of the ground, he drags himself along. This is the condition which authorizes such an expression as "the bungled and faulty composition of the sloth." But when he reaches the branch or the rough bark of a tree, his progress is rapid; he climbs hand over head along the branches till they touch, and thus from bough to bough, and from tree to tree; he is most alive in the storm, and when the wind blows, and the trees stoop, and the branches wave and meet, he is then upon the march." (P. 27.)

The third chapter, on the Comparative Anatomy of the Hand, is a very good one. After some account of the shoulder, and then of the scapula in general, we come to a few interesting observations on the situation of this bone in the horse.

"Some interest is attached to the position of the scapula, in the horse. In him, and in other quadrupeds, with the exceptions which I have made, there is no clavicle, and the connexion between the extremity and the trunk is solely through muscles. That muscle called serratus magnus, which is a large one in man, is particularly powerful in the horse; for the weight of the trunk hangs upon this muscle. In the horse, as in most quadrupeds, the speed results from the strength of the loins and hinder extremities; for it is the muscles there which propel the animal. But were the anterior extremities joined to the trunk firmly, and by bone, they could not withstand the shock from the descent of the whole weight thrown forwards; even though they were as powerful as the posterior extremities, they would suffer fracture or dislocation. We cannot but admire, therefore, the provision in all quadrupeds whose speed is great, and whose spring is extensive, that, from the structure of their bones, they have an elastic resistance, by which the shock of resistance is diminished.

"If we observe the bones of the anterior extremity of the horse, we shall see that the scapula is oblique to the chest; the humerus oblique to the scapula; and the bones of the fore-arm at an angle with the humerus. Were these bones connected together in straight lines, end to end, the shock of alighting would be conveyed through a solid column, and the bones of the foot, or the joints, would suffer from the concussion. When the rider is thrown forwards on his hands, and more certainly when he is pitched on his shoulder, the collar-bone is broken, because in man this bone forms a link of connexion between the shoulder and the trunk, so as to receive the whole shock; and the same would happen in the horse, the stag, and all quadrupeds of great strength and swiftness, were not the scapulæ sustained by muscles, and not by bone, and did not the bones recoil and fold up.

"The horse-jockey runs his hand down the horse's neck, in a knowing way, and says, 'this horse has got a heavy shoulder, he is a slow horse!' He is right, but he does not understand the matter; it is not possible that the shoulder can be too much loaded with muscle, for muscle is the source of motion, and bestows power. What the jockey feels, and forms his judgment on, is the abrupt transition from the neck to the shoulder, which, in a horse for the turf, ought to be a smooth undulating surface. This abruptness, or prominence of the shoulder, is a consequence of the upright position of the scapula; the sloping and light shoulder results from its obliquity. An upright shoulder is the mark of a stumbling horse: it does not revolve easily, to throw forward the foot." (P. 52.)

In the same chapter we find a curious zoological anecdote.

"I have alluded to the observation of President Jefferson on the Megalonix. Having found a bone, which, by its articulating surface and general form, he recognised to be one of the bones of the phalanx of an animal of great size, he thought he could discover that it had carried a claw; and from this circumstance he naturally enough concluded (according to the adage—ex ungue leonem,) that it must have belonged to a carnivorous animal. He next set about calculating the length of this claw, and estimating the size of the animal. He satisfied himself that in this bone, a relic of the ancient world, he had obtained a proof of the existence, during these old times, of a lion of the height of the largest ox, and an opponent fit to cope with the mastodon. But when this bone came under the scrutiny of Baron Cuvier, his perfect knowledge of anatomy enabled him to draw a different conclusion.

"He first observed that there was a spine in the middle of the articulating surface of the last bone; which in this respect was unlike the form of the same bone in the feline tribe. He found no provision, in this specimen of an extinct animal, from the lateral attachment of the bone; which we have just shown to be necessary for its retraction. Then observing what portion of a circle this bone formed, he prolonged the line, and showed the claw belonging to it must have been of such great length, that it could never have been retracted to the effect of guarding an acute and sharp point. The point, therefore, could not have been raised vertically, so as to have permitted the animal to put the foot to the ground without blunting the instrument! Pursuing such a comparison, he rejected the idea of the bone belonging to an animal of the feline tribe at all. His attention was directed to another order, the paresseux or sloths, which have great toes and long nails. (P. 26.) Their nails are folded up in a different fashion; they just enable the animal to walk, but slowly and awkwardly, something in the same manner as if we were to fold our fingers on the palm of the hand, and bear upon our knuckles. On instituting a more just comparison between these bones of the ancient animal and the corresponding bones of the paresseux, he has satisfied us that the lion of the American President was an animal which scratched the ground and fed on roots.

One experiences something like relief to find that there never was such an enormous carnivorous animal as this denominated megalonix." (P. 97.)

Perhaps the finest chapter in the book—the best among the good—is the seventh, *Of Sensibility and Touch.* We can indulge ourselves only with a short extract from it.

"A nerve, possessed of a quality totally different from that of the optic nerve, extends over all the exterior surfaces of the eye, and gives to those surfaces their delicate sensibility. Now it sometimes happens that this nerve is injured and its function lost, the consequences of which are very curious ; smoke and offensive particles, which are afloat in the atmosphere, rest upon the eye; flies and dust lodge under the eyelids, without producing sensation, and without exciting either the hydraulic or the mechanical apparatus to act for the purpose of expelling them. But, although they do not give pain, they nevertheless stimulate the surfaces so as to produce inflammation, and that causes opacity in the fine transparent membranes of the eye; and the organ is lost, although the proper nerve of vision remains entire. I have seen many instances of the eye being thus destroyed for want of sensibility to touch,* and it has been curious to remark, on these occasions, that when the hand was waved, or a feather brought near the eye, the person winked; yet he did not shut his eye on rubbing the finger across the eyeball, or when blood was removed by the lancet from the inflamed vessels. In those cases, when vision gave notice of danger to the organ, the patient winked to avoid it, but when the point touched the eye or eyelids, the sense of touch gave no alarm, and was followed by no action for the protection of the organ.

"I shall present another instance of the peculiar nature of the sensibility which protects the eye. The oculist has observed that

* "They are stated at length in my papers in the Philosophical Transactions, and in the Appendix of my work on the Nervous System."

by the touch of a thing as light as a feather, the muscles of the eye will be thrown into uncontrollable actions and spasms; but if the point of the finger be pressed somewhat rudely between the eyelids, and directly on the eye itself, he can by such means hold the eye steady for his intended operation, producing hardly any sensation, certainly no pain! This is one of the little secrets of the art: the oculist turns out the eyelids, and fingers the eye, in a manner which appears, at once, rude and masterly; and still the wonder grows that he can do such things with so much dexterity as to inflict no pain, when by daily experience we know that even a grain of sand in the eye will torture us. The explanation is this: the eye and eyelids are possessed of a sensibility which is adjusted to excite the action of its protecting parts against such small particles as might lodge and inflame its fine membranes. But the apparatus is not capable of protecting the surface of the eye against the intrusion of a stick or a stone; from such injuries it could not be defended by a delicate sensibility and involuntary action, but only by the effort of the will." (P. 163.)

In the following exquisite sentences, which we take from the "concluding remarks," one of the evils attending the progress of refinement is beautifully depicted: instead of feeling, we analyze;—a rose becomes a thing to be studied in a lecture-room, and the moon is to be seen—nicely mapped out in the Penny Magazine!

"We may consider man, before the lights of modern philosophy had their influence on his thoughts, as in a state more natural; inasmuch as he yielded unresistingly to those sentiments which directly flow from the objects and phenomena around him. But when that period of society arrived in which man made natural phenomena the subjects of experiment or of philosophical inquiry, then was there some danger of a change of opinion, not always beneficial to his state of mind. This danger does not touch the philosopher so much as the scholar. He who has strength of mind and ingenuity to make investigations into nature will not be satisfied with the discovery of secondary causes; his mind will be enlarged, and the objects of his thoughts and aspirations become more elevated. But it is otherwise with those not themselves habituated to investigation, and who learn at second-hand the result of those inquiries. If such a one sees the fire of heaven brought down into a phial, and materials compounded to produce an explosion louder than the thunder, and ten times more destructive, the storm will no longer speak an impressive language to him. When, in watching the booming waves of a tempestuous sea, along the coast, he marks the line at which the utmost violence of the ocean is stemmed, and by an unseen influence thrown back, he is more disposed to feel the providence extended to man, than when the theory of the moon's action is as it were interposed between the scene which he contemplates and the sentiments natually arising in his breast. Those influences on the mind which are natural, and just, and beneficently provided, and have served to develope the sentiments of millions before him, are dismissed as things vulgar and to be despised. With all the pride of newly-acquired knowledge, his conceptions embarrass, if they do not mislead him; in short, he has not had that intellectual discipline which should precede and accompany the acquisition of knowledge." (P. 229.)

We must close our extracts, though reluctantly, with our author's observations on the Sus Ethiopicus; they are in the additional illustrations.

"When we look upon the boar's head, we comprehend something of his habits, and see what must be the direction of his strength. He feeds by digging up roots, and the instruments by which he does this are also those of his defence. The position of the tusk defends the eye in rushing through the underwood; and the formation of the skull and of the spine, and the mass of muscle in the neck, all shew the intention that he shall drive onward with his whole strength, so that he may rend with his tusks. Accordingly, we may see that the back part of the skull rises in remarkable spines or ridges for the attachment of muscles, and that, corresponding with these, the spinous processes of the vertebræ of the neck and back are of extraordinary length and strength. These processes distinctly indicate the power of the muscles which pass from the neck to the head. We now understand the reason of the shortness and inflexibility of the neck; because the power of the shoulders is directed to the head, and, we may say, to these large tusks. An elongated and flexible neck would have rendered these provisions useless. The characteristic form of the wild boar, then, consists in the height of the back, the shortness and thickness of the neck, the wedge shape of the head, the projection of the tusk, and the shortness of the fore legs, which must always be in proportion to the neck." (P. 257.)

These copious quotations will enable our readers to form some conception of the grace and suavity which breathe through this excellent treatise. Nor are the illustrations inferior to the text. Our author's skill and taste as an artist enable him to illustrate his works with unusual elegance. The figures in the present volume are drawn with Sir Charles Bell's wonted felicity; and, in many instances, the *chiaro oscuro* has been made out with a richness and effect which until lately were thought to be the exclusive and enviable privilege of copper-plate engravings. Yet there are one or two cases in which the neatness of the drawing must not prevent our noticing the incorrectness of the fact which it is intended to represent.

At page 103 there is a sketch of the head, chest, and anterior member of a dolphin. The scapula is here represented in a false position, its base being made vertical to the spine, instead of nearly parallel to it. If the scapula of the dolphin were really placed in the supposed position, the fin which it supports would strike downwards exclusively, instead of backwards, and, instead of aiding the progression of the animal, would serve only to assist its rise towards the surface. We conjecture that our author was misled in this instance by considering the direction of the tail in the cetaceous mammalia.

At page 57 a figure is introduced of the scapula and fore leg of an elephant, which are contrasted with those of the camel; and while, in the latter, the ordinary flexion of the shoulder-joint and elbow of a quadruped is preserved, in the former the elbow is represented in a straight position, the direction given to the arm-bone is vertical, and the scapula is disposed vertically upon it. Sir Charles Bell supposes that the greater weight of the elephant renders this position of the bones necessary, in order that the muscles may be spared, as much as possible, the fatigue of supporting so enormous a mass. The fact however is, that the direction of the shoulder-blade, humerus, and fore-arm, are the same in the elephant and in the camel; and the frame, in both instances, is equally balanced upon muscular springs. The skeleton of Chuny, now preserved in the museum of the College of Surgeons, must not be cited against us, for its articulation is erroneous. The bones of Chuny were set up by a Manchester shoemaker, who saw that in this mercantile age he might interpose himself with advantage as middleman between the menagerist and the philosopher.

We now take leave, but we trust only for a short time, of Sir Charles Bell; a man in whom the utmost facility in acquiring knowledge is almost surpassed by the ardent zeal with which he imparts it.

